# A diatom/PdNPs nanocomposite carbon paste electrode for sensitive voltammetric determination of aloe emodin: electrodeposition, characterization, and oxidation mechanism

**DOI:** 10.1039/d6ra06249k

**Published:** 2026-09-02

**Authors:** Buse İrtiş, Zehra Yazan, Ceren Yıldız, Aysel Koç Demir, Dilek Eskiköy Bayraktepe, Kamran Polat

**Affiliations:** a Department of Chemistry, Faculty of Science, Ankara University Ankara Türkiye zdurmus@science.ankara.edu.tr zehrayazan67@gmail.com

## Abstract

Aloe emodin (AE, 1,8-dihydroxy-3-(hydroxymethyl)anthraquinone) is a naturally occurring anthraquinone derivative with well-documented anticancer, antiviral, and anti-inflammatory properties, making it an important target for sensitive analytical determination. In this study, a novel electrochemical sensor based on a diatom/palladium nanoparticle nanocomposite-modified carbon paste electrode (DA/PdNPs/CPE) was developed for the highly sensitive and selective determination of AE in herbal samples. The carbon paste electrode was first modified with purified diatomaceous earth (DA), and palladium nanoparticles (PdNPs) were subsequently electrodeposited onto the DA/CPE surface *via* chronoamperometry (CA) to yield the composite sensing platform. The fabricated electrode was thoroughly characterized by cyclic voltammetry (CV), electrochemical impedance spectroscopy (EIS), and field-emission scanning electron microscopy coupled with energy-dispersive X-ray spectroscopy (FE-SEM/EDS), confirming enhanced electrical conductivity, reduced charge-transfer resistance, and uniform deposition of PdNPs across the electrode surface. Under optimized conditions, square-wave voltammetry (SWV) was employed for the quantitative determination of AE, yielding a linear response in the concentration ranges of 0.05–2.0 µM and 2.0–8.0 µM with a limit of detection (LOD) of 0.0064 µM and a limit of quantification (LOQ) of 0.0213 µM. The sensor demonstrated excellent precision (RSD = 2.52%) and reproducibility (RSD = 1.35%). The practical applicability of the sensor was validated by determining AE in a herbal vitamin supplement, with recovery values ranging from 100.17% to 100.50%, thereby confirming its suitability for real-sample analysis. Furthermore, the electrooxidation mechanism of AE on the modified electrode surface was investigated and detailed.

## Abstract 2

In this study, a novel electrochemical sensor based on a diatom/palladium nanoparticle nanocomposite-modified carbon paste electrode (DA/PdNPs/CPE) was developed for the highly sensitive and selective determination of AE in herbal samples.

## Graphical abstract

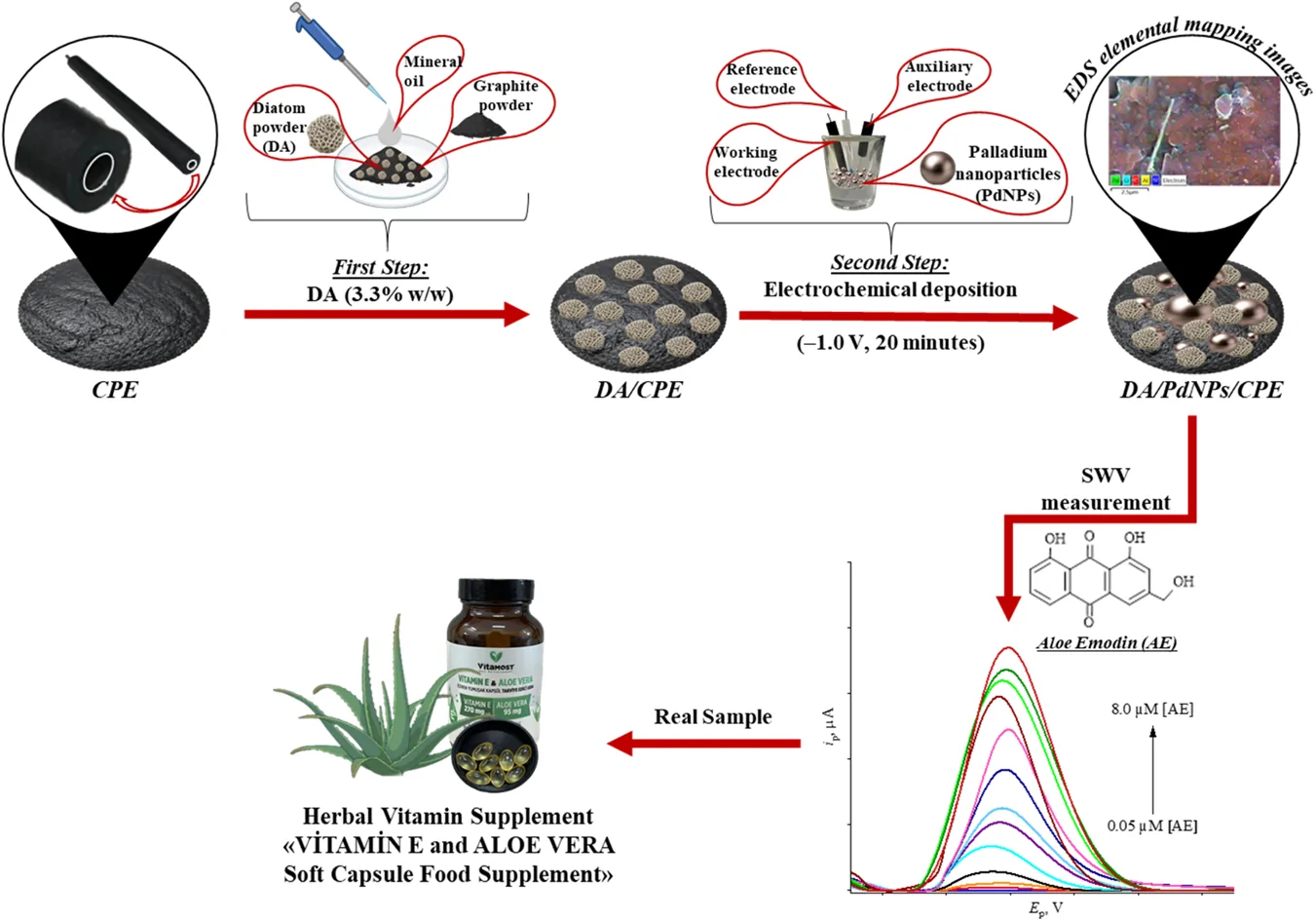

## 1. Introduction

Aloe emodin (AE, 1,8-dihydroxy-3-(hydroxymethyl)anthraquinone) is a naturally occurring anthraquinone derivative classified among polyphenolic compounds. It is found in the gel, sap, and leaves of various medicinal plants, including *Aloe vera*, *Cassia occidentalis*, *Rheum palmatum* L., and *Polygonum multiflorum* Thunb. Numerous pharmacological studies have demonstrated that AE exhibits anticancer, antiviral, anti-inflammatory, and antimicrobial activities,^1–3^ and it is also widely employed in cosmetic formulations as an emollient, moisturizer, and cicatrisant.^4^ Furthermore, AE has been reported to induce apoptosis in several tumor cell lines, including lung carcinoma, hepatocellular carcinoma, and promyelocytic leukemia cells.^5–8^ At the molecular level, AE inhibits kinase activity by binding to mTORC2, thereby suppressing cell proliferation and triggering apoptosis.^9,10^ However, high-dose or prolonged exposure to AE has been associated with adverse effects, including phototoxicity, hepatotoxicity, and nephrotoxicity.^11^

Given its significant biological activity and potential toxicity, accurate quantitative determination of AE in pharmaceutical formulations, biological fluids, and cosmetic products is of considerable importance. Several analytical methods have been developed for this purpose, including UV spectrophotometry,^12^ fluorimetry,^13,14^ high-performance liquid chromatography (HPLC),^15,16^ gas chromatography-mass spectrometry (GC-MS),^17^ capillary electrophoresis,^18^ and electrochemical approaches.^19–21^ Among these, electroanalytical techniques have attracted considerable attention owing to their high sensitivity, selectivity, short analysis time, low cost, and good reproducibility. Compared to conventional instrumental methods, electrochemical approaches offer additional advantages, including minimal sample pretreatment, straightforward operation, and accessibility without requiring highly specialized instrumentation.^22,23^

Various electrode materials have been employed in electrochemical sensing, including glassy carbon electrodes (GCE), carbon paste electrodes (CPE), and pencil graphite electrodes (PGE), each offering distinct surface properties and electrochemical potential windows suited to specific applications. Among these, CPEs are particularly favored due to their low background current, wide potential window, chemical inertness, low fabrication cost, high sensitivity, good reproducibility, and ease of surface modification.^24,25^ To further enhance the analytical performance of CPEs, surface modification with nanomaterials has been widely explored. In particular, noble metal nanoparticles—including Au, Ag, Pt, Pd, and Ru—have attracted growing interest owing to their unique electrical, catalytic, and optical properties.^26^ Among these, palladium nanoparticles (PdNPs) have emerged as particularly promising modifiers due to their lower cost relative to Pt, superior resistance to CO poisoning, excellent electrical conductivity, large specific surface area, and strong affinity for biological molecules.^27,28^

Diatoms are a diverse group of microalgae widely distributed across marine, freshwater, and terrestrial environments. Their silica frustules exhibit a highly ordered three-dimensional nanostructure with inherent thermal and mechanical stability, biocompatibility, and characteristic optical properties.^29,30^ In electrochemical sensing, diatomaceous earth—a porous, silica-rich material derived from fossilized diatom frustules—has been used as an electrode modifier to enhance sensitivity and charge-storage capacity.^31^ The abundant reactive Si–OH groups on the diatom surface promote strong interfacial interactions and facilitate uniform coating on electrode substrates. Incorporation of diatomaceous earth into nanocomposite electrode materials has been shown to improve electrochemical performance by enhancing structural integrity, chemical durability, and overall sensor stability.^32,33^

In the present study, a novel electrochemical sensor based on the combination of palladium nanoparticles and purified diatomaceous earth was developed for the sensitive determination of AE. The carbon paste electrode was first modified with purified diatomaceous earth, and PdNPs were subsequently electrodeposited onto the DA/CPE surface by chronoamperometry to yield the DA/PdNPs/CPE sensing platform. The composite electrode was characterized by FE-SEM, EDS, CV, and EIS to investigate its morphological, compositional, and electrochemical properties. To the best of our knowledge, this is the first report of a PdNPs/diatom-based electrochemical sensor for AE determination, providing a simple, selective, and low-cost platform for its quantitative analysis. The fabricated sensor was further evaluated for AE determination in real samples. Although Pd nanoparticles have been employed as electrode modifiers in various electrochemical sensing platforms, and diatom earth has been explored as a natural support material, the combination of these two components has not been reported for the determination of AE or, to the best of our knowledge, for any anthraquinone-based analyte. The proposed DA/PdNPs/CPE platform is fabricated entirely from commercially available, low-cost materials through a straightforward two-step procedure, eliminating the need for complex chemical synthesis, template removal, or expensive precursor materials required by molecularly imprinted polymer- or graphene-based sensors.

## 2. Materials and methods

### 2.1 Reagents, solutions, and apparatus

Graphite powder, mineral oil, potassium hexacyanoferrate(ii) trihydrate (K_4_[Fe(CN)_6_]·3H_2_O), potassium hexacyanoferrate(iii) (K_3_[Fe(CN)_6_]), sodium phosphate dibasic (Na_2_HPO_4_), sodium dihydrogen phosphate (NaH_2_PO_4_), palladium(ii) chloride (PdCl_2_), and all solvents were purchased from Sigma-Aldrich (St. Louis, MO, USA). Aloe emodin (AE) standard was also obtained from Sigma-Aldrich. Moreover, interference species include standard solutions of calcium (Ca^2+^), magnesium (Mg^2+^), zinc (Zn^2+^), and copper (Cu^2+^) in 3% HNO_3_ at 1000 mg L^−1^, supplied by Merck, Darmstadt, Germany, along with dopamine (DOPA), uric acid (UA), ascorbic acid (AA), NaHCO_3_, and KCl, which were purchased from Sigma-Aldrich. Raw natural diatom samples (rDA) were provided by the BENTAŞ firm (Kazan, Ankara, Türkiye). We prepared a 1.0 mM stock solution of AE by dissolving an accurately weighed amount in ethanol and stored it at +4 °C until analysis. The pH of the supporting electrolyte was adjusted with 0.1 M NaOH or 0.1 M HCl. A standard solution of 5.0 mM [Fe(CN)_6_]^3−/4−^ redox probe was prepared by dissolving equimolar amounts of K_3_[Fe(CN)_6_] and K_4_[Fe(CN)_6_] in 0.1 M KCl solution. All other chemicals were of analytical reagent grade and used without further purification. Double-distilled deionized water (resistivity ≥ 18.2 MΩ cm) was obtained from an MPminipure Ultra-Pure Water System (MES, Turkey) and used throughout the experiments.

We performed electrochemical measurements, including CV, SWV, and EIS, using a PalmSens4 portable potentiostat/galvanostat with a C3 cell stand (PalmSens BV, Netherlands). We acquired and processed all electrochemical data using PSTrace 5.9 software. A conventional three-electrode cell configuration was employed, consisting of bare CPE, PdNPs/CPE, DA/CPE, or DA/PdNPs/CPE as the working electrode, Ag/AgCl (in 3.0 M KCl, BASi MW-1032) as the reference electrode, and a platinum wire (BASi MF-2052) as the auxiliary electrode. We conducted pH measurements using a Thermo Scientific Orion Star A111 pH meter, calibrated with standard buffer solutions at pH 4.0, 7.0, and 10.0 before each measurement.

Surface morphology and elemental composition of the modified electrodes were characterized by FE-SEM coupled with EDS using a Hitachi SU5000 instrument operated at 15 kV. Elemental analysis of diatom samples was performed using an X-ray fluorescence (XRF) spectrometer (Rigaku ZSX Primus II) equipped with a Pd/Co alloy anode X-ray tube (50 W, 60 kV). Particle size distributions were determined using a Malvern Mastersizer 3000 laser diffraction particle size analyzer.

All electrochemical measurements were carried out at room temperature (25 ± 2 °C) unless otherwise stated.

### 2.2 Purification of diatom

Raw natural diatom samples (rDA) provided by the BENTAŞ firm (Kazan, Ankara, Türkiye) were initially crushed and sieved through a 100-mesh sieve to obtain a uniform particle size. The sieved diatomaceous earth powder was pre-washed with deionized water to remove surface impurities, such as dust and stone fragments, and then decanted. Subsequently, the samples were treated with 5 M HCl under continuous stirring at 500 rpm for 12 h at room temperature to dissolve inorganic impurities, including metal oxides and carbonates. After the acid treatment, the suspension was allowed to settle overnight. The solid phase was then separated by filtration and washed repeatedly with deionized water until chloride ions were completely removed, as confirmed by the AgNO_3_ precipitation test. Finally, the purified solid was dried in an oven at 60 °C for 24 h and subsequently calcined in air at 550 °C for 3 h to remove residual organic matter. The chemical composition and physicochemical properties of both rDA and DA samples were characterized using X-ray fluorescence (XRF) spectroscopy and laser diffraction particle size analysis, respectively.

### 2.3 Fabrication of the DA/PdNPs/CPE sensor

The fabrication of the modified electrode involved two sequential steps:

In the first step, we prepared a diatom-modified carbon paste electrode (DA/CPE). Purified diatom powder (DA, 1.0 mg, 3.3% w/w), graphite powder, and mineral oil (10 µL) were combined to a total mass of 30 mg and thoroughly mixed in an agate mortar using a pestle for 5 min to obtain a homogeneous paste. The paste was packed into a cylindrical PTFE tube body, and electrical contact was established *via* a copper wire inserted into the electrode's rear end. The exposed electrode surface was polished on weighing paper until it achieved a mirror-like finish.

In the second step, palladium nanoparticles (PdNPs) were electrodeposited onto the DA/CPE surface by chronoamperometry at a constant applied potential of −1.0 V (*vs.* Ag/AgCl) for 20 minutes in 0.1 M PBS (pH 7.0) containing 0.01 µM PdCl_2_. The resulting DA/PdNPs/CPE was thoroughly rinsed with double-distilled water to remove unbound precursor species and dried at room temperature prior to use.

### 2.4 Electrochemical procedure

The solutions for electrochemical studies were prepared as follows:

A 0.05 M PBS solution was titrated with 0.1 M HCl to adjust the pH to 7.0, then a calculated volume of stock AE solution was added to reach a predetermined analyte concentration, bringing the total volume to 10 mL. This solution was transferred to the voltammetric cell and used for SWV and CV measurements. The voltammograms were recorded in the potential range of (−0.75)–(−0.15) V. The SWV conditions were given as follows: amplitude: 0.025 V, step potential: 0.02 V, frequency: 5.0 Hz.

### 2.5 Herbal vitamin supplement sample tablet preparation

For the herbal vitamin supplement sample preparation, five capsules were selected to obtain a reliable mean value. The outer gelatin shells were carefully pierced, and the liquid contents were completely withdrawn using a syringe and combined in a single vessel to prepare a homogeneous stock solution. The AE concentration in this stock solution was approximately 2.1 µM. We precisely transferred a 50.0 µL aliquot of the stock solution into the electrochemical cell containing the PBS supporting electrolyte. Quantification was performed by the standard addition method, and all measurements were carried out in triplicate (*n* = 3). The endogenous AE concentration in the sample was determined from the intercept of the standard addition plot, and the recovery was calculated as the ratio of the measured to the added AE concentration, expressed as a percentage.

## 3. Results and discussion

### 3.1 Characterization of diatom

XRF analysis revealed that the relative silicon (Si) content of the purified diatom (DA) increased compared to the raw sample (rDA) (Table S1), consistent with an enrichment of the silica matrix following removal of accessory mineral phases. This increase is attributed to the dissolution and removal of co-existing impurity phases rather than an absolute gain in silica mass. Notably, the concentrations of aluminum (Al), magnesium (Mg), iron (Fe), barium (Ba), and sodium (Na) all decreased after purification. Collectively, these results confirm that the acid washing and calcination procedure effectively purified the diatom framework, yielding a silica-enriched product suitable for electrode modification.

The particle size distribution analysis shows that raw diatom (rDA) has a notably wide, dispersed distribution across the 11–70 µm range (Fig. 1). After purification, the distribution becomes more concentrated within a specific interval. Furthermore, the proportion of large particles (50–70 µm) in the rDA decreased significantly above approximately 44 µm after purification, dropping to near-zero levels around 70 µm. These data indicate that large-grained impurities and aggregates were effectively removed from the structure during the purification process. Additionally, the purification process resulted in a shift toward a narrower particle-size distribution.

**Fig. 1:**
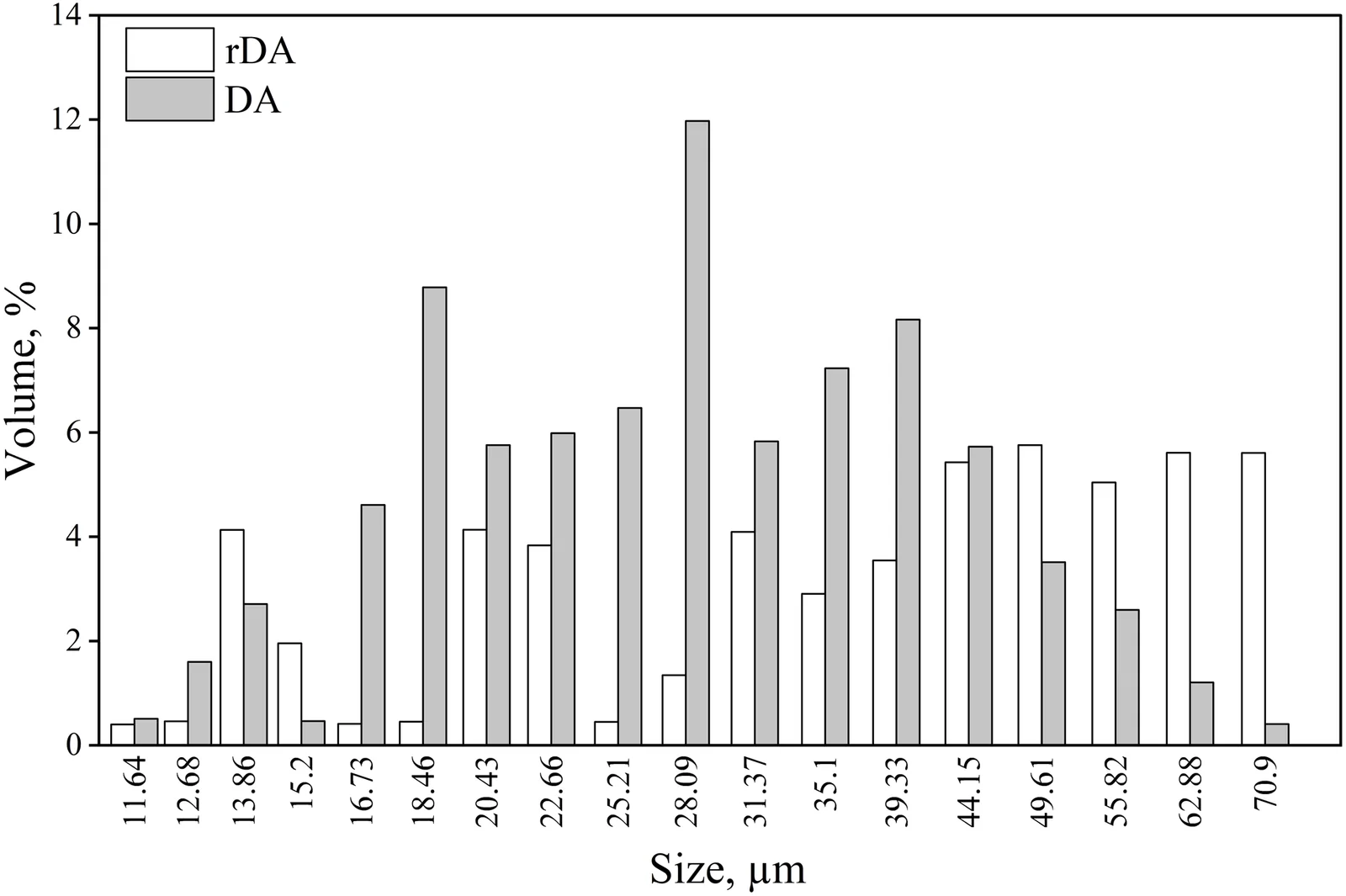
Particle size distribution graph of rDA and DA.

### 3.2 Electrochemical characterization of the DA/PdNPs/CPE sensor

The electrochemical characterization of bare CPE, PdNPs/CPE, DA/CPE, and DA/PdNPs/CPE was investigated by CV and EIS using a 5.0 mM [Fe(CN)_6_]^3−/4−^ redox probe in 0.1 M KCl. Fig. 2a presents the CVs recorded at a scan rate of 50 mV s^−1^. The bare CPE exhibited the lowest anodic and cathodic peak currents, while stepwise modification with DA, PdNPs, and finally DA/PdNPs progressively enhanced both peak currents of the [Fe(CN)_6_]^3−/4−^ redox couple. The DA/PdNPs/CPE yielded the highest peak currents among all tested electrodes. Furthermore, the bare CPE displayed the largest peak-to-peak separation (Δ*E*_p_ ≈ 700 mV), whereas the DA/PdNPs/CPE exhibited the smallest Δ*E*_p_ (≈420 mV), indicating faster electron transfer kinetics at the composite-modified surface. These findings confirm the cooperative electrocatalytic contribution of DA and PdNPs: PdNPs enhance electrical conductivity and promote electron transfer, while the diatom's porous silica framework provides a high surface area that facilitates analyte adsorption and accumulation at the electrode interface.

**Fig. 2:**
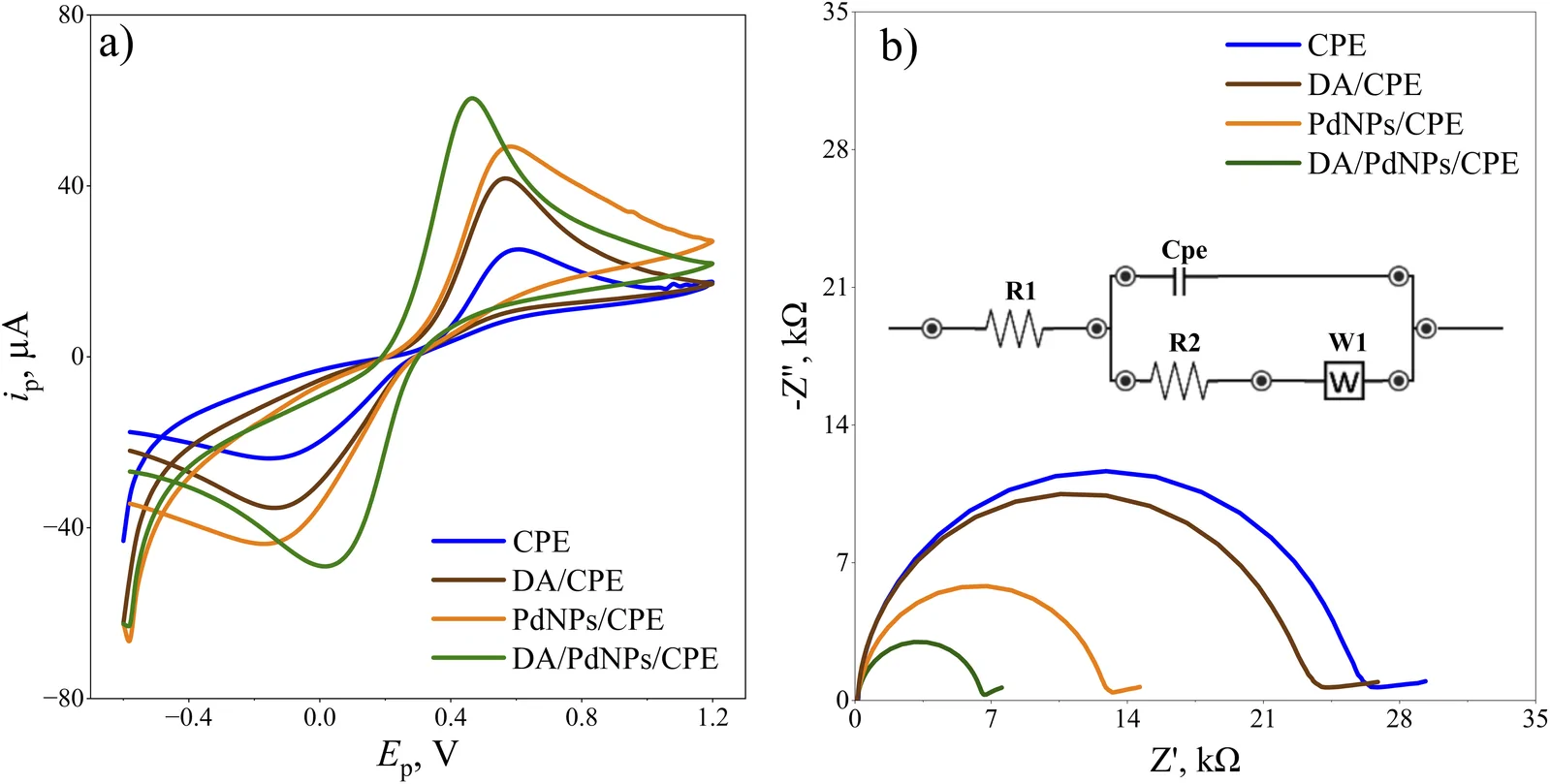
Electrochemical characterization of bare CPE, DA/CPE, PdNPs/CPE, and DA/PdNPs/CPE in 5.0 mM [Fe(CN)_6_]^3−/4−^ containing 0.1 M KCl: (a) cyclic voltammograms recorded at a scan rate of 50 mV s^−1^ and (b) Nyquist plots obtained by EIS.

The EIS technique was used to obtain further information about electron transfer and the conductive surface of the modified electrodes. In general, the Nyquist plot in EIS consists of two parts: a linear region indicating a diffusion-controlled process at low frequencies and a semicircle corresponding to an electron-transfer-controlled process at high frequencies. The EIS Nyquist plots (Fig. 2b) further support the CV observations in the same medium, with the semicircle diameter decreasing progressively upon modification and reaching its smallest value at DA/PdNPs/CPE.

The impedance data were fitted using the equivalent circuit shown in the inset of Fig. 2b, consisting of the solution resistance (R1), a constant phase element (Cpe) accounting for the non-ideal capacitive behavior of the rough carbon paste surface, the charge-transfer resistance (R2), and the Warburg impedance (W1) associated with diffusion of the redox probe. Table S2 summarizes the fitted parameters. The charge-transfer resistance decreased markedly upon modification, from 25.28 kΩ for bare CPE to 22.76 kΩ for DA/CPE, 12.74 kΩ for PdNPs/CPE, and 6.36 kΩ for DA/PdNPs/CPE, corresponding to a four-fold reduction and confirming substantially enhanced electron-transfer kinetics at the composite-modified surface. In parallel, the Cpe magnitude increased from 1.26 × 10^−7^ to 3.62 × 10^−7^ S s^*n*^, consistent with the enlarged electroactive surface area determined independently by the Randles–Ševčík analysis, while the Warburg coefficient decreased from 1315 to 392 Ω s^−1/2^, indicating facilitated mass transport within the porous composite structure. All fits yielded reduced chi-square values below 2.56 × 10^−4^, confirming the adequacy of the selected circuit model. These EIS results agree well with the CV data and collectively demonstrate successful fabrication of the composite-modified electrode.

The electrochemically active surface areas of bare CPE, DA/CPE, PdNPs/CPE, and DA/PdNPs/CPE were calculated using the Randles–Ševčík equation under non-standard conditions for quasi-reversible electrochemical processes.^34^ CV voltammograms were recorded in 0.1 M KCl containing 5.0 mM [Fe(CN)_6_]^3−/4−^ at a scan rate of 50 mV s^−1^, and the measurements were repeated (*n* = 3) for each electrode surface. We calculated the results using *n* = 1 and a diffusion coefficient of 7.6 × 10^−6^ cm^2^ s^−1^ for [Fe(CN)_6_]^3−/4−^; the reported standard deviations correspond to replicate measurements. The active surface area (*A*) values of bare CPE, DA/CPE, PdNPs/CPE, and DA/PdNPs/CPE were calculated to be 0.0320 (±0.0004) cm^2^, 0.0462 (±0.0003) cm^2^, 0.0531 (±0.0005) cm^2^, and 0.0642 (±0.0007) cm^2^, respectively. The DA/PdNPs/CPE exhibited an approximately 2.0-fold higher active surface area compared to bare CPE, indicating that the incorporation of diatom and palladium nanoparticles significantly enhances the active surface area and electrical conductivity of the composite-modified electrode.

### 3.3 Morphological and compositional characterization of the DA/PdNPs/CPE sensor

For the morphological characterization, FE-SEM images and corresponding EDS spectra of bare CPE, DA/CPE, PdNPs/CPE, and DA/PdNPs/CPE are presented in Fig. 3.

**Fig. 3:**
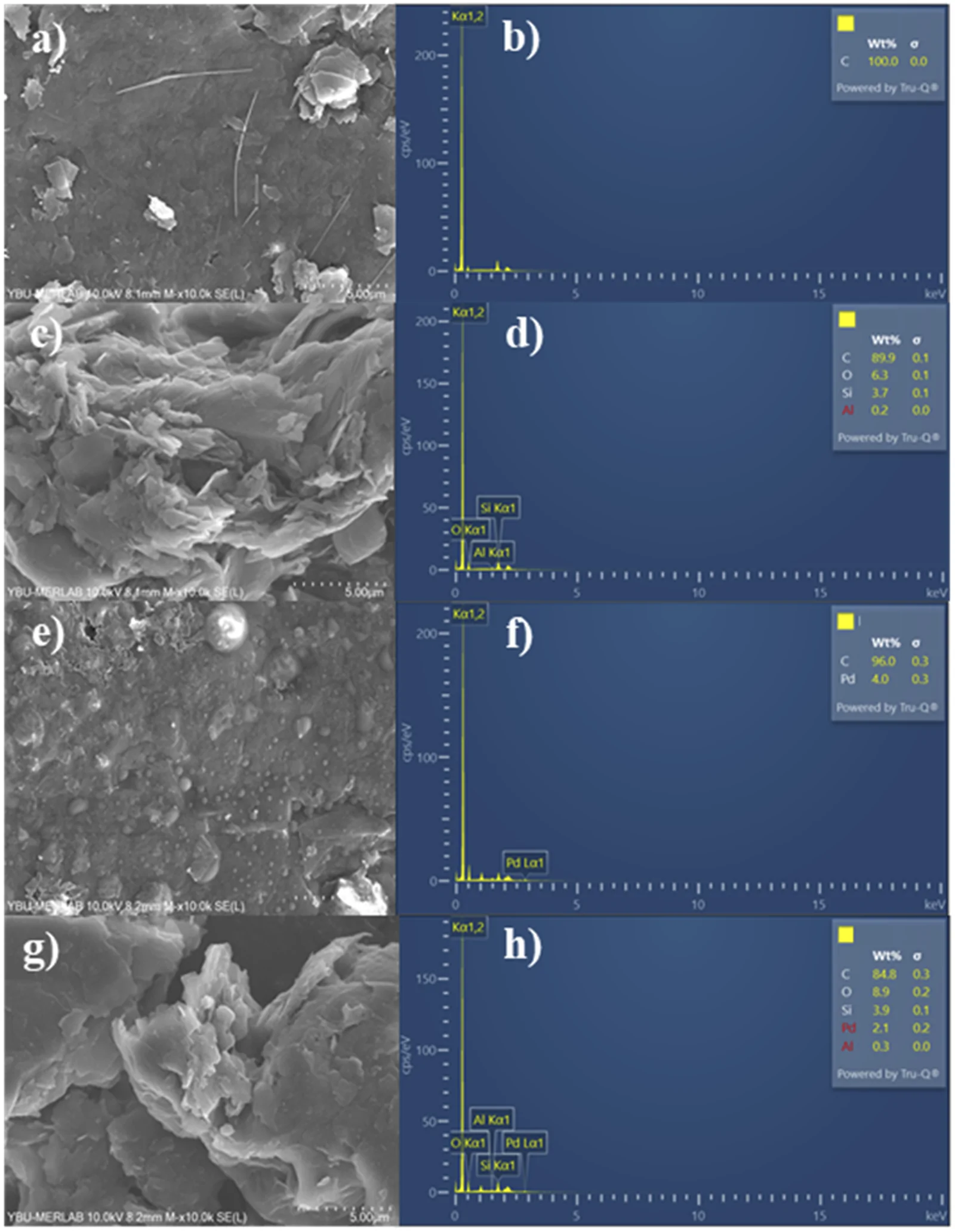
FE-SEM images and corresponding EDS spectra of (a) and (b) bare CPE, (c) and (d) DA/CPE, (e) and (f) PdNPs/CPE, and (g) and (h) DA/PdNPs/CPE.

The bare CPE surface (Fig. 3a) exhibited a relatively smooth morphology with layered graphite flakes, and the corresponding EDS spectrum (Fig. 3b) confirmed a pure carbon composition (C: 100 wt%) with no detectable heteroatoms, thereby verifying the chemical purity of the unmodified electrode.

Following diatom incorporation (DA/CPE, Fig. 3c), the surface morphology changed markedly, revealing an irregular, rough texture characteristic of the diatom silica frustule. The EDS spectrum (Fig. 3d) confirmed the presence of silicon (Si: 3.7 wt%), oxygen (O: 6.3 wt%), and aluminum (Al: 0.2 wt%) alongside carbon (C: 89.9 wt%), consistent with the aluminosilicate composition of diatoms and confirming successful incorporation of DA into the carbon paste matrix.

The PdNPs/CPE surface (Fig. 3e) exhibited uniformly distributed spherical nanoparticles throughout the graphite matrix, indicating successful electrodeposition of palladium nanoparticles *via* chronoamperometry. The EDS spectrum (Fig. 3f) confirmed the presence of Pd (4.0 wt%) alongside C (96.0 wt%), with no other elemental contributions detected, demonstrating the selective deposition of PdNPs onto the CPE surface.

The DA/PdNPs/CPE (Fig. 3g) exhibited a complex surface morphology combining the rough diatom framework with spherical PdNPs distributed across the surface. The EDS spectrum (Fig. 3h) confirmed the coexistence of all expected elements: C (84.8 wt%), O (8.9 wt%), Si (3.9 wt%), Pd (2.1 wt%), and Al (0.3 wt%), thereby confirming the successful fabrication of the composite-modified electrode. The simultaneous detection of Si and Pd signals provides direct chemical evidence for the co-existence of the diatom silica matrix and palladium nanoparticles on the electrode surface.

To further investigate the elemental distribution on the DA/PdNPs/CPE surface, EDS elemental mapping was performed, as shown in Fig. 4. The mapping images confirm the presence and distribution of all expected elements—carbon (C, Fig. 4b), oxygen (O, Fig. 4c), silicon (Si, Fig. 4e), aluminum (Al, Fig. 4a), and palladium (Pd, Fig. 4d)—across the electrode surface. The carbon signal is dominant and uniformly distributed, consistent with the graphite paste matrix. The oxygen, silicon, and aluminum signals, though heterogeneously distributed, are spread across the surface, confirming the successful incorporation of DA into the carbon paste. Notably, the Si mapping (Fig. 4e) reveals a distinct elongated feature, likely corresponding to an individual diatom frustule, superimposed on a more diffuse background signal. The Pd signal (Fig. 4d), while relatively low in intensity, is distributed across the surface, confirming the electrodeposition of PdNPs onto the electrode. The composite elemental overlay (Fig. 4f) and the corresponding EDS spectrum (Fig. 4g) collectively confirm the co-existence of all five elements, with weight percentages of C (84.8%), O (8.9%), Si (3.9%), Pd (2.1%), and Al (0.3%), demonstrating the successful fabrication of the DA/PdNPs/CPE nanocomposite electrode.

**Fig. 4:**
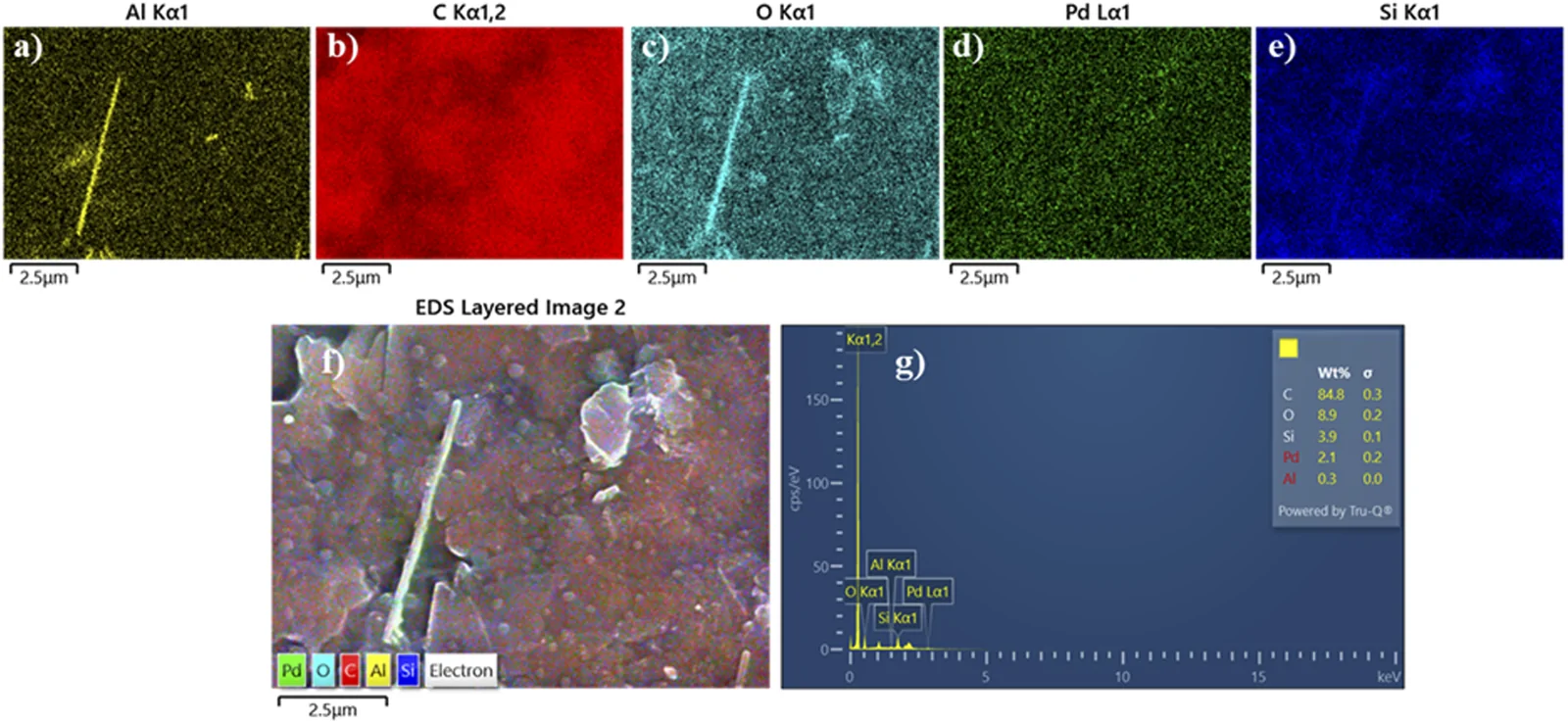
EDS elemental mapping images of DA/PdNPs/CPE: (a) Al, (b) C, (c) O, (d) Pd, and (e) Si distribution maps; (f) composite overlay of all elements; (g) corresponding EDS spectrum with elemental weight percentages.

### 3.4 Comparative electrochemical performance of CPE sensors

Following surface characterization, the electrocatalytic performance of each electrode was evaluated by recording SWV responses toward 0.50 µM AE in 0.05 M PBS (pH 7.0) (Fig. 5). The bare CPE exhibited an AE oxidation peak at approximately −0.46 V with the lowest peak current among all tested surfaces. Upon modification, the oxidation peak potential shifted by approximately 0.04 V toward less positive values, reaching −0.50 V at DA/PdNPs/CPE, indicating a reduction in the overpotential required for AE oxidation and confirming the electrocatalytic nature of the composite surface. The distinct contributions of the two modifiers are evident from the stepwise enhancement: DA/CPE alone provides a 2.6-fold increase attributable to enhanced surface area and adsorptive accumulation of AE within the porous diatom framework, while PdNPs/CPE provides a 3.8-fold increase from improved electron-transfer kinetics. Their combination in DA/PdNPs/CPE yields a 5.4-fold enhancement, indicating that the two modifiers contribute cooperatively through complementary mechanisms. The outstanding performance of DA/PdNPs/CPE is attributed to the cooperative contribution of the high-surface-area diatom framework, which promotes analyte accumulation, and the PdNPs, which enhance electron-transfer kinetics. Consequently, DA/PdNPs/CPE was selected as the optimal sensing platform for subsequent studies.

**Fig. 5:**
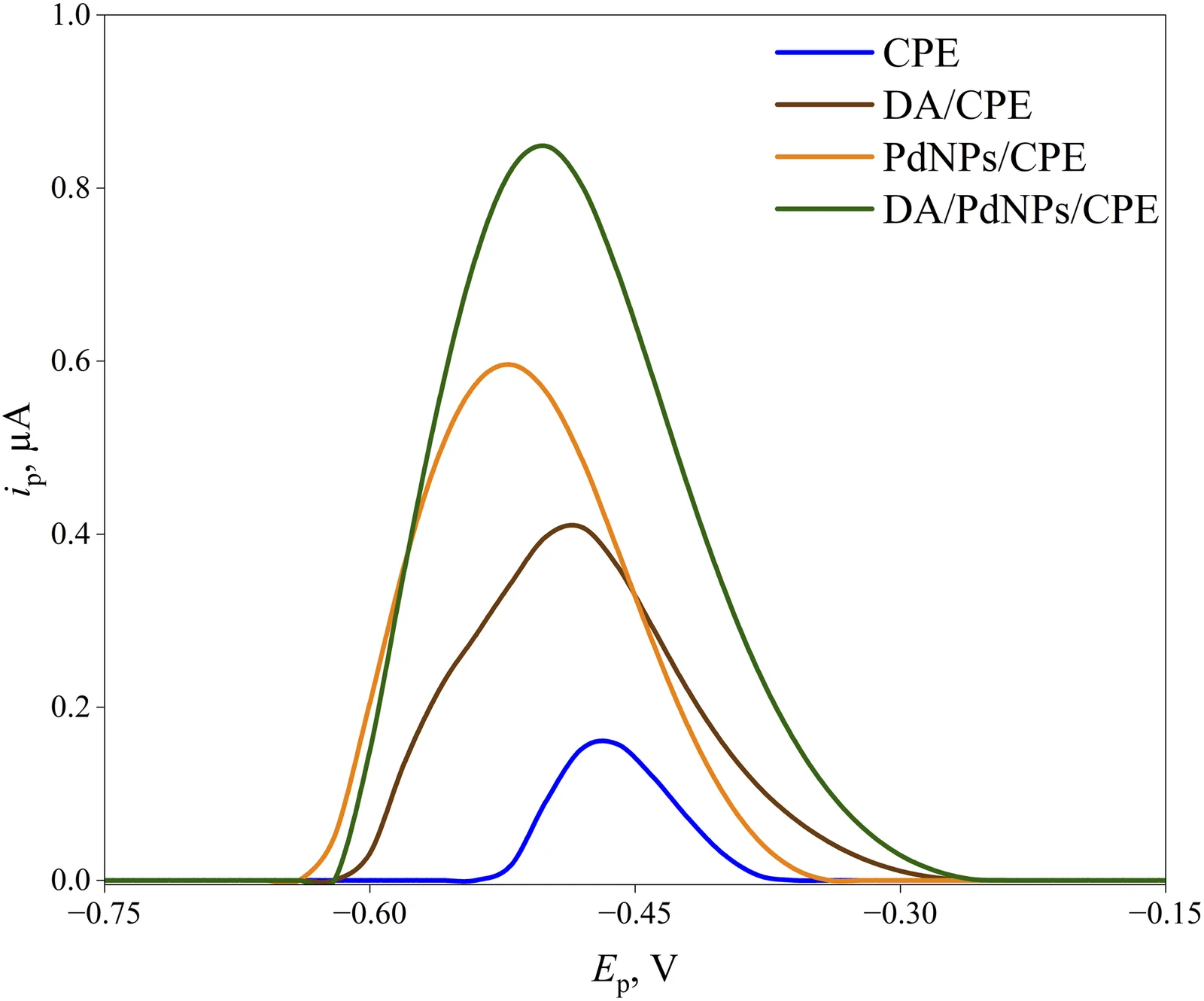
SWV responses of 0.50 µM AE recorded at bare CPE, DA/CPE, PdNPs/CPE, and DA/PdNPs/CPE in 0.05 M PBS (pH 7.0).

### 3.5 Optimization of analytical and sensor parameters for AE detection

After identifying DA/PdNPs/CPE as the optimal sensor platform, the sensor fabrication parameters were systematically optimized. First, the effect of DA content in the carbon paste was investigated by varying the DA-to-paste ratio (0.5/30, 1/30, 1.5/30, 2/30, and 3/30, w/w) while keeping all other conditions constant. SWV responses toward 0.5 µM AE were recorded in 0.05 M PBS (pH 7.0) for each composition. As shown in Fig. S1a, the highest anodic peak current was obtained at a DA ratio of 1/30 (3.3% w/w, corresponding to 1.0 mg DA per 30 mg paste with 10 µL mineral oil), beyond which the signal decreased. This behavior reflects the dual role of the diatom content: up to 3.3% (w/w), the porous silica framework increases the effective surface area and provides adsorption sites that promote AE accumulation at the electrode interface, whereas at higher loadings the insulating silica begins to disrupt the continuity of the conductive graphite network, reducing the electrical conductivity of the paste and hindering electron transfer. Therefore, 3.3% (w/w) DA was selected as the optimal paste composition.

Subsequently, the effect of PdCl_2_ concentration on the electrodeposition of PdNPs was examined by varying the concentration from 0.005 to 0.7 µM (Fig. S1b). The maximum peak current was observed at 0.01 µM; further increases led to a decline in signal. Up to 0.01 µM, increasing the PdCl_2_ concentration enhances the density of electrocatalytically active Pd sites on the electrode surface; at higher concentrations, however, the deposited nanoparticles tend to grow and aggregate, decreasing the effective electroactive surface area and consequently the sensor response. Accordingly, a PdCl_2_ concentration of 0.01 µM was selected as the optimal condition for PdNP deposition.

Finally, the effect of Pd deposition time on the DA/CPE surface, measured by chronoamperometry, on the AE oxidation signal was investigated (Fig. S1c). The peak current increased from 5 to 20 min and decreased thereafter. Prolonged deposition results in excessive Pd loading, which blocks the pores of the diatom framework and limits analyte accessibility to the underlying active sites, thereby reducing the effective electroactive surface area available for AE oxidation.

The effect of solution pH on the electrochemical oxidation of AE was investigated using SWV in 0.05 M PBS over the pH range of 6.0–8.0 (Fig. S2). A solution of 0.5 µM AE was prepared in 0.05 M PBS at each pH value, and the peak current of AE was measured on the DA/PdNPs/CPE surface. As shown in Fig. S2a, the anodic peak current reached its maximum at pH 7.0, whereas lower and higher pH values resulted in a progressive decrease in signal intensity. Accordingly, pH 7.0 was selected as the optimal supporting electrolyte pH for subsequent experiments. In Fig. S2b, the observed peak potential shift with increasing pH suggests that protons participate in the electrochemical oxidation of AE at the electrode surface, consistent with a proton-coupled electron-transfer process commonly reported for hydroxyl-substituted anthraquinone derivatives.^19^

### 3.6 Electrochemical behavior of AE

The main voltammetric methods used to elucidate the organic compounds' redox behavior are CV, SWV, and differential pulse voltammetry (DPV) at the interface of electrode/solution. Polyphenols undergo a common electrochemical oxidation at the –OH groups, which is affected by chemical substituents on aromatic rings (*e.g.*, –OCH_3_, –CH_3_). Experimental conditions, such as pH, directly affect the redox behavior of polyphenols and the formation of oxidation products.^35^ Oxidation reactions can also be followed by chemical reactions in which the oxidation product and/or the resulting dimers and polymers may be adsorbed onto the electrode surface. To gain an insight into the nature of the electrode process, the effect of scan rate (*ν*) on the CV response of AE was investigated over the range of 0.050–0.350 V s^−1^ (Fig. S3). The anodic peak current (*i*_p_) increased linearly with both *ν* and *ν*^1/2^ (Fig. S3a and c), while the log *i*_p_–log *ν* plot (Fig. S3b) yielded a slope of 0.6856 (*R*^2^ = 0.9864), a value intermediate between the theoretical slopes expected for purely diffusion-controlled (0.5) and purely adsorption-controlled (1.0) processes. This result indicates that the electrode reaction of AE at the DA/PdNPs/CPE surface proceeds *via* a mixed diffusion–adsorption-controlled mechanism, consistent with the high surface area and adsorptive character of the diatom framework, which promotes accumulation of AE and its oxidation products at the electrode interface.

The number of protons involved in the electrochemical process was investigated over the pH range of 6.0–8.0 (Fig. S2b). The SWV curves indicated that the AE peak potential at the modified electrode shifted toward more negative values as pH increased. This indicated that proton transfer was involved in the electrode reaction. The relationship between the anodic peak potential (*E*_pa_) and pH was found to be *E*_pa_ = −0.045 pH + 0.186 (*R*^2^ = 0.9888). The slope of −0.045 for the oxidation peak is close to the theoretical Nernstian value of −0.059.^36,37^ This result demonstrates that the transferred numbers of electrons and protons are equal in the oxidation mechanism of AE.

To conclude the detailed oxidation mechanism of AE, the number of electrons transferred in the oxidation process was calculated by eqn (1) given below for irreversible reactions:^37^1
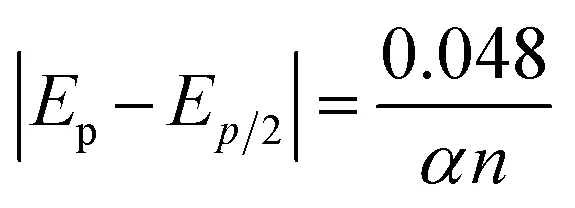
Here, *n* is the number of electrons transferred, α is the electron transfer coefficient assumed to be 0.5 for a totally irreversible electrode process,^38^*E*_p_ is the peak potential, and *E*_p/2_ is the half-peak potential. Based on eqn (1), the number of transferred electrons was calculated to be approximately 1.0, consistent with a one-electron oxidation step. Electrochemical studies on the oxidation of AE showed one electron/one proton, corresponding to the irreversible oxidation of AE to the semiquinone structure, along with C–C coupling, at a potential of about −0.45 V.^39,40^ The electrooxidation mechanism, followed by chemical reaction steps at pH 7.0, is shown in Scheme 1.

**Scheme 1:**
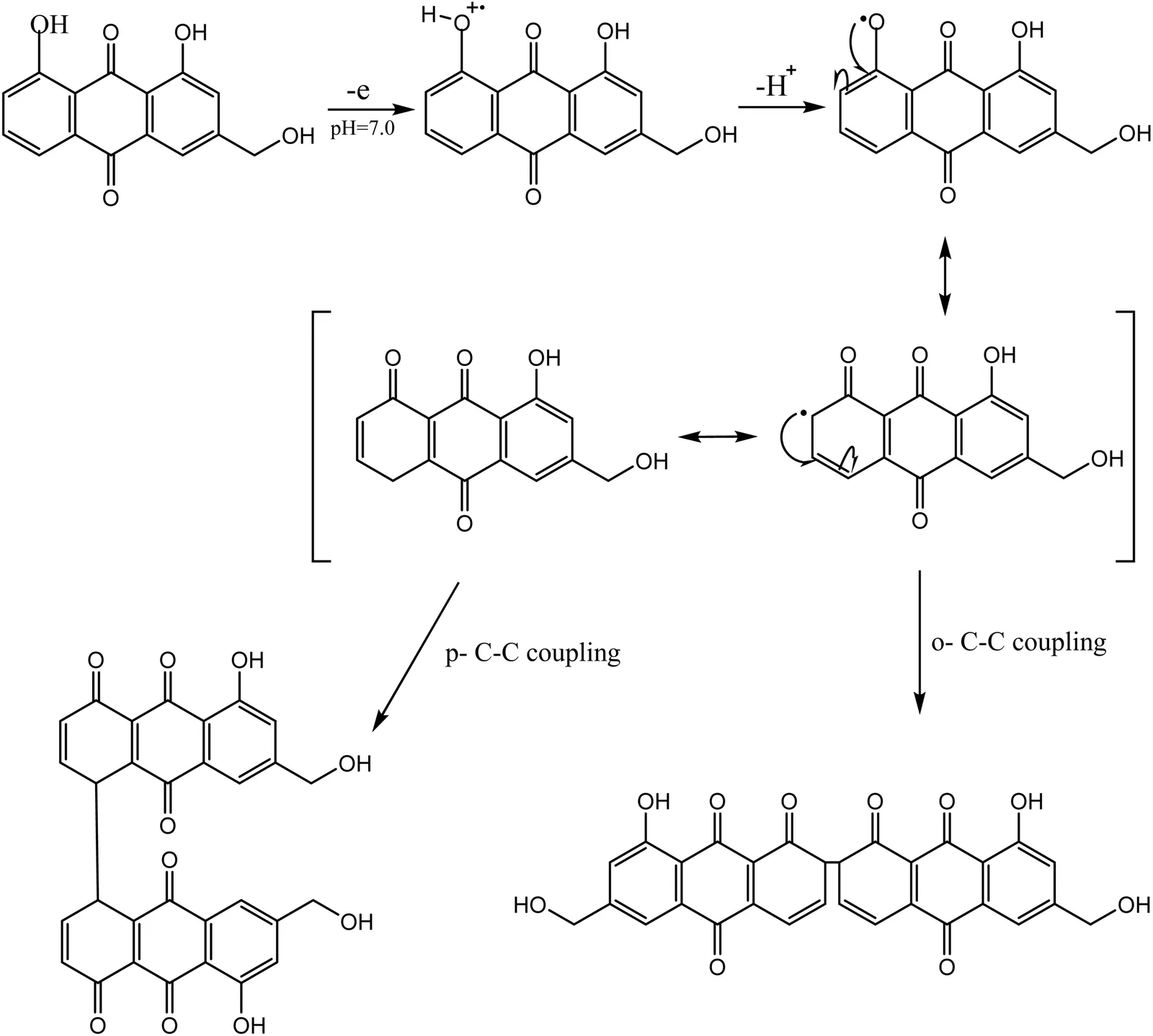
Tentative electrooxidation mechanism of AE in PBS buffer solution (pH 7.0) on DA/PdNPs/CPE.

The proposed mechanism is supported by several complementary experimental observations: the pH dependence of the peak potential (−0.045 V pH^−1^, Fig. S2b) confirms proton participation; the calculated electron number (*n* ≈ 1) indicates a one-electron transfer step; the log *i*_p_–log *ν* slope of 0.6856 (Fig. S3b) reflects a mixed diffusion–adsorption-controlled process consistent with adsorption of oxidation products at the electrode surface; and the absence of a corresponding cathodic peak in cyclic voltammetry confirms an irreversible electrochemical step followed by a chemical reaction (EC mechanism). This mechanistic framework, based on one-electron oxidation of the phenolic hydroxyl group followed by radical–radical C–C coupling, is well established for hydroxyl-substituted anthraquinones and is therefore expected to be applicable to structurally related derivatives such as emodin and chrysophanol, although the regioselectivity of the coupling step may vary with the substitution pattern.

### 3.7 Calibration studies

The analytical performance of the DA/PdNPs/CPE sensor was evaluated under optimized conditions using SWV (Fig. 6). The anodic peak current increased linearly with AE concentration over two dynamic ranges: 0.05–2.0 µM and 2.0–8.0 µM, with regression coefficients of *R*^2^ = 0.9944 and *R*^2^ = 0.9983, respectively (inset of Fig. 6). The LOD and LOQ were calculated using the expressions LOD = 3*s*/*m* and LOQ = 10*s*/*m*,^41^ yielding values of 0.0064 µM and 0.0213 µM, respectively, confirming the high sensitivity of the proposed sensor. Table 1 summarizes all regression parameters.

**Fig. 6:**
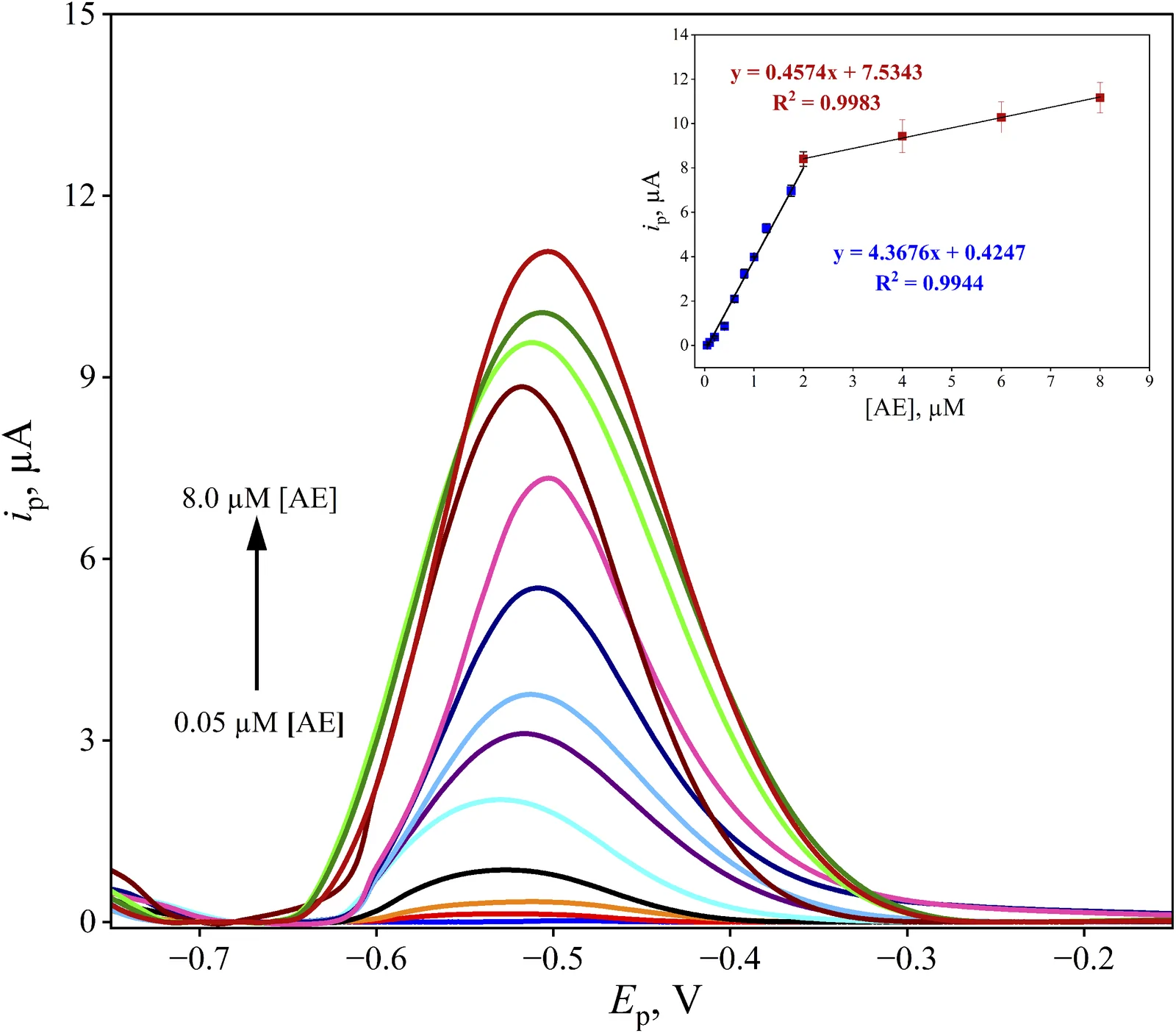
SWVs recorded at DA/PdNPs/CPE in 0.05 M PBS (pH 7.0) with increasing concentrations of AE. Inset: Linear calibration plot of anodic peak current (*i*_pa_, µA) as a function of AE concentration (µM). SWV parameters: pulse time: 0.1 s, amplitude: 25 mV, step potential: 20.0 mV, scan rate: 100 mV s^−1^.

**Table 1:** Regression parameters and validation data for the electrochemical determination of AE at DA/PdNPs/CPE

Parameters	AE
Linear working range (µM)	0.05–2.0; 2.0–8.0
Regression coefficient (*R*^2^)	0.9944; 0.9983
Limit of detection, LOD (µM)	0.0064
Limit of quantification, LOQ (µM)	0.0213
Intra-day precision, RSD%^a^ (peak current)	2.52
Intra-day precision, RSD%^a^ (peak potential)	1.37
Intermediate precision, RSD%^a^ (peak current)	5.47
Intermediate precision, RSD%^a^ (peak potential)	2.43
Reproducibility, RSD%^a^ (peak current)	1.35
Reproducibility, RSD%^a^ (peak potential)	0.98

a RSD values were calculated from five replicate measurements (*n* = 5) for precision and five independently fabricated electrodes (*n* = 5) for reproducibility. LOD = 3*s*/*m*; LOQ = 10*s*/*m*, where *s* is the standard deviation of the blank, and *m* is the slope of the calibration curve.

The occurrence of two distinct linear ranges is characteristic of electrode processes involving analyte adsorption. At lower AE concentrations (0.05–2.0 µM), abundant active sites are available within the three-dimensional porous diatom framework, allowing efficient accumulation of AE at the electrode surface and resulting in a steeper calibration slope (4.3676 µA µM^−1^). As the concentration increases beyond 2.0 µM, the available adsorption sites become progressively saturated, and the electrode response becomes increasingly governed by diffusion of AE from the bulk solution, producing a markedly lower slope (0.4574 µA µM^−1^). This interpretation is supported by the scan-rate study (Fig. S3b), in which the log *i*_p_–log *ν* plot yielded a slope of 0.6856, a value intermediate between those expected for purely diffusion-controlled (0.5) and purely adsorption-controlled (1.0) processes, confirming that the electrode reaction of AE at DA/PdNPs/CPE proceeds *via* a mixed diffusion–adsorption-controlled mechanism.

The precision of the sensor was assessed by intra-day measurements of 0.5 µM AE on the same electrode, yielding an RSD of 2.52% for peak current. The intermediate precision, evaluated over three consecutive days, gave an RSD of 5.47%. Reproducibility was examined using five independently fabricated electrodes of the same composition. The RSD value for peak current reproducibility (1.35%) further confirmed the precision and reliability of the fabricated sensor. All RSD values were within acceptable limits, demonstrating satisfactory precision for analytical applications, as summarized in Table 1.

Table 2 presents a performance comparison of the developed DA/PdNPs/CPE sensor with sensors reported in the literature. As summarized in Table 2, numerous electrochemical platforms have been developed for AE determination, ranging from bare GCE,^42,43^ and CPE^44^ to nanomaterial-modified,^21,45^ and molecularly imprinted polymer (MIP)-based electrodes.^20,46^ While MIP-based sensors offer selectivity advantages, their fabrication involves complex synthesis and template removal steps that compromise reproducibility. Nanomaterial-modified GCE platforms^21^ have achieved lower LOD values but require expensive modifiers and multi-step preparation protocols. Among CPE-based sensors specifically, the previously reported platforms—including bare CPE,^44^ ionic liquid-type CPIE,^19^ and TiO_2_/CPE^47^—exhibited either limited linear ranges or significantly higher LOD values (up to 1.89 µM (ref. 47)), underscoring the superior sensitivity and broader dynamic range achieved by the DA/PdNPs nanocomposite modification strategy proposed in this work. In contrast, the DA/PdNPs/CPE sensor proposed herein combines a naturally abundant, low-cost diatom modifier with electrodeposited PdNPs *via* a simple, reproducible fabrication procedure. The proposed sensor achieved a competitive LOD of 0.0064 µM, a wide working range of 0.05–2.0 µM and 2.0–8.0 µM, and excellent recovery values of 100.17–100.50% in real herbal vitamin supplement samples. To the best of our knowledge, this represents the first application of a diatom/PdNPs nanocomposite-modified CPE for AE detection, offering a simple, low-cost, and reliable alternative to existing methods.

**Table 2:** Comparison of voltammetric methods reported for the electrochemical determination of AE^a^

Electrode	Method	Analyte	Linear range (µM)	LOD (µM)	Real sample	Ref.
CPIE	DPV	AE	0.01–12.4	0.003	Standard sample with AE	19
PMM-MIP@G	DPV	AE	0.0005–350	0.0003	AE in cosmetic samples	20
GN/GC	DPV	AE	0.005–1.0	0.002	Natural aloe extracts and human urine samples	21
GCE	CV	AE	0.089–7.8	0.0078	Anticancer herbal drug	42
GCE	DASV	AE	0.2–8.0	0.1	AE sample (juice, skin, gel, and whole leaf)	43
CPE	LSV/CV	AE	0.0001–0.04	0.00004	Radix Rhei extract with AE	44
CNN/GCE	SWV	AE	0.00624–1.13; 1.13–12.3	2.08	Standard sample with AE	45
MIP/CNPs/GCE	DPV	AE	0.1–100.0	0.075	Cassia seed, capsule aloes composite, and aloe vera gel	46
TiO_2_/CPE	SWV	AE	5.0–150.0	1.89	Real flotation and synthetically contaminated soil samples	47
Pt electrode	DPV	AE	1.0–150.0	0.2	—	48
Pd AuNp/Pr/GE	AdsSWV	AE	0.036–0.08; 0.09–0.3	0.011	Aloe vera capsule	49
	EIS		0.059–0.6	0.015		
**DA/PdNPs/CPE**	SWV	AE	0.05–2.0; 2.0–8.0	0.0064	Herbal vitamin supplement	**This work**

a CPIE: ionic liquid-type carbon paste electrode; PMM-MIP@G: graphite-based molecularly imprinted poly(methyl methacrylate) carbon paste electrode; GN/GC: graphene-Nafion modified glassy carbon electrode; CNN/GCE: carbon-coated nickel magnetic nanoparticles modified glassy carbon electrode; CPE: carbon paste electrode; MIP/CNPs/GCE: molecularly imprinted polymer and carbon nanoparticles modified glassy carbon electrode; Pt electrode: platinum electrode; TiO_2_/CPE: titanium dioxide modified carbon paste electrode; GCE: glassy carbon electrode; Pd AuNp/Pr/GE: palladium–gold bimetallic nanoparticle and polypropylene modified graphite electrode; AE: aloe emodin; DPV: differential pulse voltammetry; CV: cyclic voltammetry; AdSV: adsorptive stripping voltammetry; LSV: linear sweep voltammetry; SWV: square wave voltammetry; AdsSWV: square wave adsorptive stripping voltammetry; EIS: electrochemical impedance spectroscopy.

### 3.8 Interferences

The selectivity of the DA/PdNPs/CPE sensor was evaluated by examining how potential interfering species affected the SWV response of 0.1 µM AE in 0.05 M PBS (pH 7.0). We tested the following common coexisting species at molar ratios of 1 : 1, 1 : 10, and 1 : 100 (AE : interferent): Ca^2+^, Mg^2+^, Zn^2+^, Cu^2+^, dopamine (DOPA), uric acid (UA), ascorbic acid (AA), NaHCO_3_, and KCl. As shown in Fig. S4 and summarized in Table S3, none of the tested species caused a significant change in the anodic peak current of AE at 1 : 1 and 1 : 10 concentration levels, with all relative errors remaining within +6.0%. These results demonstrate that the proposed sensor exhibits high selectivity toward AE in the presence of common interfering substances, confirming its reliability for analytical determination in complex sample matrices.

### 3.9 Real sample analysis

The practical applicability of the DA/PdNPs/CPE sensor was assessed by determining AE in commercially available soft gel capsules containing aloe vera extract (vitamin E and aloe vera soft capsule food supplement). To eliminate potential matrix interferences arising from the complex composition of the herbal extract, the standard addition method was employed for quantification. Known concentrations of AE standard solution (0.4, 0.6, and 0.8 µM) were successively spiked into the sample solution in the electrochemical cell, and the corresponding SWV responses were recorded after each addition. The resulting calibration plot yielded a regression coefficient (*R*^2^) of 0.9981, confirming a highly linear sensor response to increasing AE concentrations in the real sample matrix. The recovery values ranged from 100.17% to 100.50%, demonstrating the proposed method's excellent accuracy. Table 3 summarizes the complete recovery data and RSD% values. These results confirm that the developed sensor can reliably determine AE in herbal vitamin supplement samples with complex excipient matrices, without significant matrix interference.

**Table 3:** Recovery results for the determination of AE in the herbal vitamin supplement sample by the standard addition method (*n* = 3)

Real sample	Sample	Sample amount, µL	Added amount, µM	Found amount, µM	Average recovery, %	RSD, %
Herbal vitamin supplement (vitamin E and aloe vera soft capsule food supplement)	AE	50.0	0.40	0.402 ± 0.0061	100.50 ± 1.52	1.51
			0.60	0.601 ± 0.0091	100.17 ± 1.53	1.53
			0.80	0.803 ± 0.004	100.38 ± 0.49	0.50

### 3.10 Limitations of the present study

While the present study demonstrates satisfactory short-term stability and reproducibility, it did not evaluate long-term storage stability over several weeks, which remains to be addressed in future work. In addition, validation against a chromatographic reference method (*e.g.*, HPLC) was beyond the scope of this work, and we demonstrated analytical applicability using a single commercial herbal supplement matrix. Further studies involving a broader range of sample matrices and cross-validation with established analytical techniques would strengthen the practical applicability of the proposed sensor. Furthermore, the selectivity study did not include structurally related hydroxyanthraquinones such as emodin and chrysophanol, which may exhibit similar oxidation potentials; discriminating these compounds represents an important direction for future work.

## 4. Conclusions

In this study, we successfully developed a novel electrochemical sensor based on a DA/PdNPs nanocomposite-modified carbon paste electrode for the sensitive and selective determination of aloe emodin (AE) in herbal vitamin supplement samples. The cooperative combination of the three-dimensional porous diatom framework, which enhanced analyte adsorption and increased the effective surface area, with the electrocatalytic activity of PdNPs resulted in a markedly improved electrochemical oxidation response of AE by enhancing electron-transfer kinetics at the modified electrode surface. Under optimized conditions in 0.05 M PBS (pH 7.0), the sensor exhibited two linear dynamic ranges of 0.05–2.0 µM and 2.0–8.0 µM, with an LOD of 0.0064 µM, excellent precision (RSD = 2.52%), and high selectivity toward AE in the presence of common interfering species. The oxidation peak observed at approximately −0.50 V is tentatively attributed to the oxidation of the hydroxyl (–OH) group in the anthraquinone ring, and a detailed electrooxidation mechanism is proposed accordingly. The sensor's practical applicability was confirmed by successfully determining AE in commercial aloe vera soft gel capsules using the standard addition method, with recovery values ranging from 100.17% to 100.50%. To the best of our knowledge, this represents the first application of a DA/PdNPs-modified CPE for AE detection, offering a simple, low-cost, and reliable platform for routine quality control of herbal pharmaceutical products.

## Author contributions

Buse İrtiş: methodology, investigation, software, data curation, funding acquisition. Zehra Yazan: conceptualization, investigation, supervision, writing, review & editing. Ceren Yıldız: software, methodology, investigation, visualization. Aysel Koç Demir: data curation, methodology, software, writing – review & editing. Dilek Eskiköy Bayraktepe: data curation, investigation, methodology, writing – review & editing, writing – original draft. Kamran Polat: data curation, investigation, writing – review & editing.

## Conflicts of interest

The authors declare no conflict of interest.

## Supplementary Material

RA-016-D6RA06249K-s001

## Data Availability

Data will be made available on request. Supplementary information (SI) is available. See DOI: https://doi.org/10.1039/d6ra06249k.
