# Supplementary material for: A diatom/PdNPs nanocomposite carbon paste electrode for sensitive voltammetric determination of aloe emodin: electrodeposition, characterization, and oxidation mechanism
Source: RSC Adv. 2026 Sep 2;16(43):52531–42. doi: 10.1039/d6ra06249k (PMC13535771; doi:10.1039/d6ra06249k)
Supplement: Supplementary file 1 [file RA-016-D6RA06249K-s001.pdf]

Supplementary Information (SI) for RSC Advances.
This journal is © The Royal Society of Chemistry 2026

Supporting Information

A Diatom/PdNPs Nanocomposite Carbon Paste Electrode for Sensitive Voltammetric Determination of Aloe Emodin: Electrodeposition, Characterization, and Oxidation Mechanism

Buse İRTİŞ [a], Zehra YAZAN *[a], Ceren YILDIZ [a], Aysel KOÇ DEMİR [a], Dilek ESKİKOY BAYRAKTEPE [a] and Kamran POLAT [a]

[a] Department of Chemistry, Faculty of Science, Ankara University, Ankara, Türkiye

* Corresponding author
E-mail address: zdurmus@science.ankara.edu.tr; zehrayazan67@gmail.com (Z. Yazan)

Contents	Page
**Table S1**	2
**Table S2**	2
**Fig. S1**	2
**Fig. S2**	3
**Fig. S3**	3
**Fig. S4**	4
**Table S3**	4

Table S1 XRF elemental composition of raw diatom (rDA) and purified diatom (DA), expressed in ppm.

	Silicon	Aluminium	Iron	Magnesium	Sodium	Barium
**rDA**	356367	59230	19147	23565	10785	387
**DA**	405491	46487	16605	14832	9837	56

Table S2 Equivalent circuit parameters obtained by fitting the EIS data of bare CPE, DA/CPE, PdNPs/CPE, and DA/PdNPs/CPE using the R1 + [Cpe $_{\parallel}$ (R2 + W1)] model.

Electrode	R1 (Ω)	R2 (kΩ)	Cpe (S s^n)	n	σ (Ω $s^{-1/2}$)	χ^2_red	Residual (%)
**CPE**	151.8	25.28	1.26×10^{-7}	0.961	1315	2.56×10^{-4}	1.56
**DA/CPE**	129.8	22.76	1.51×10^{-7}	0.967	1341	2.42×10^{-4}	1.52
**PdNPs/CPE**	112.2	12.74	2.74×10^{-7}	0.942	457	1.49×10^{-4}	1.19
**DA/PdNPs/CPE**	123.5	6.36	3.62×10^{-7}	0.958	392	1.03×10^{-4}	0.99

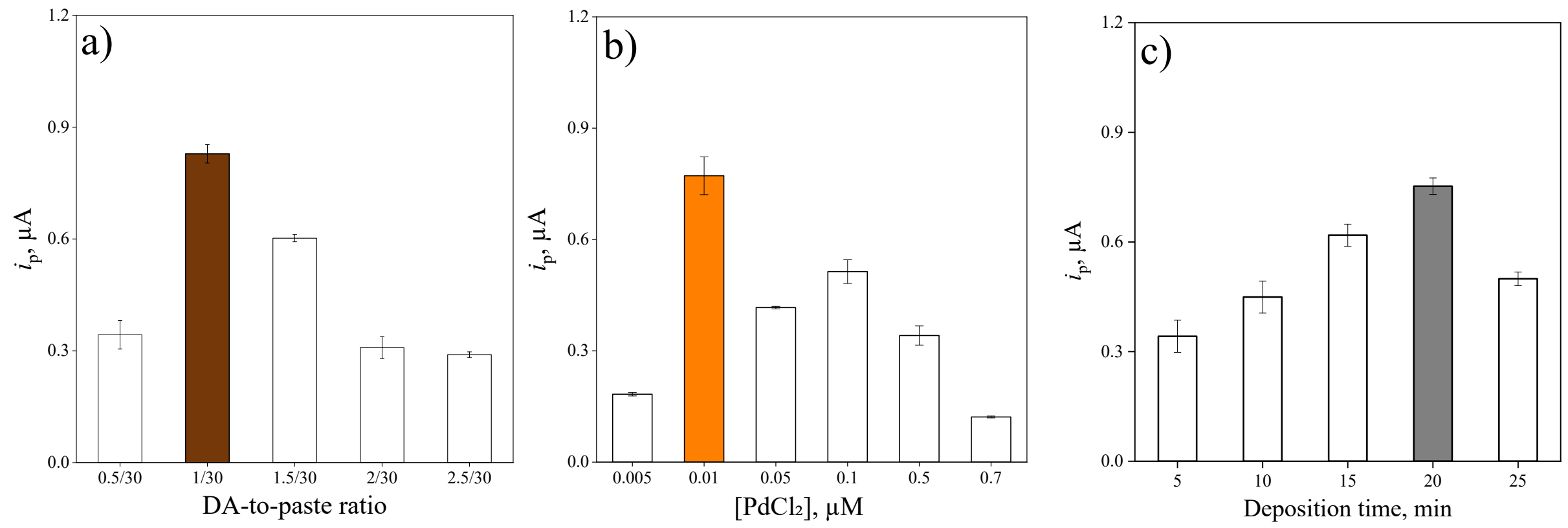

Fig. S1 The effect of (a) DA-to-paste ratio, (b) $PdCl_2$ solution concentration, and (c) deposition time on the peak current responses of 0.5 µM AE.

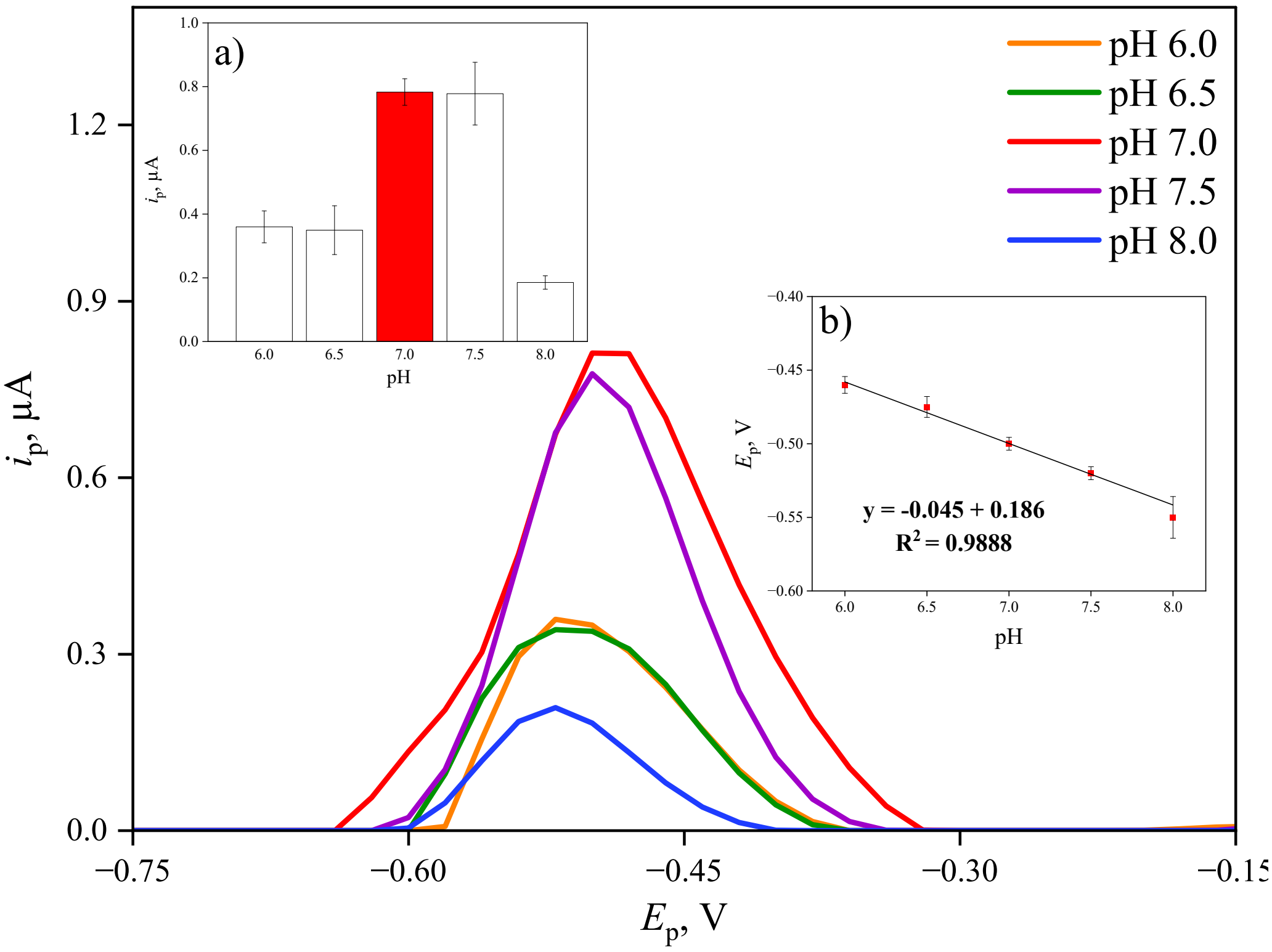

Fig. S2 Effect of pH on the SWV response of 0.5 µM AE at DA/PdNPs/CPE in 0.05 M PBS (pH 7.0). **Inset:** a) variation of anodic peak current and b) anodic peak potential as a function of pH (n = 3, error bars represent ± SD).

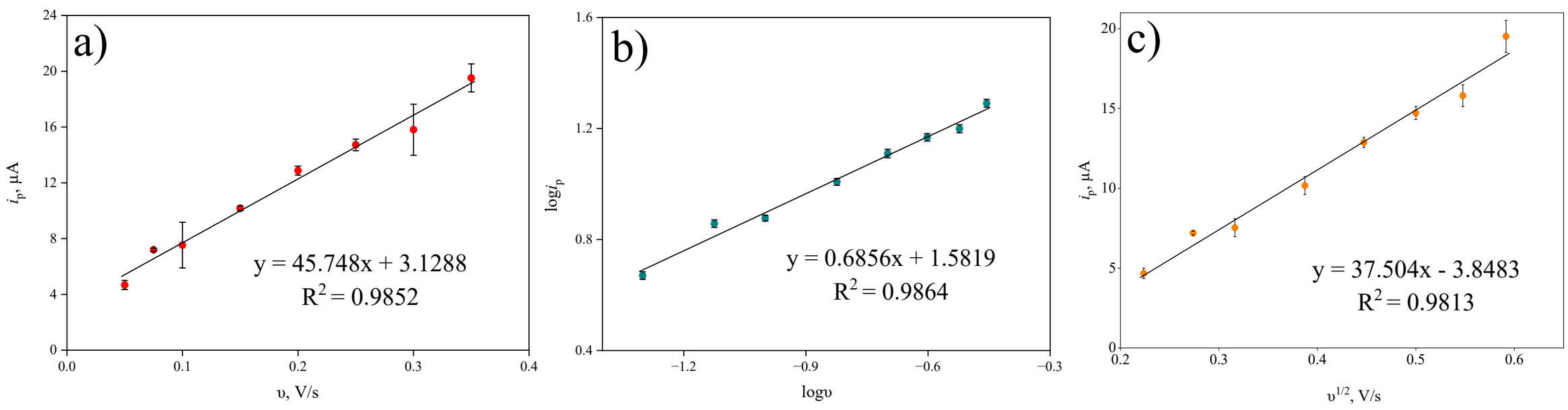

Fig. S3 100 µM AE on DA/PdNPs/CPE at scan rates ranging from 0.050 to 0.350 V/s using the CV method: a) i_p-υ, b) logi_p-logυ, and c) i_p-$υ^{1/2}$ graphs (pH 7.0, 0.05 M PBS).

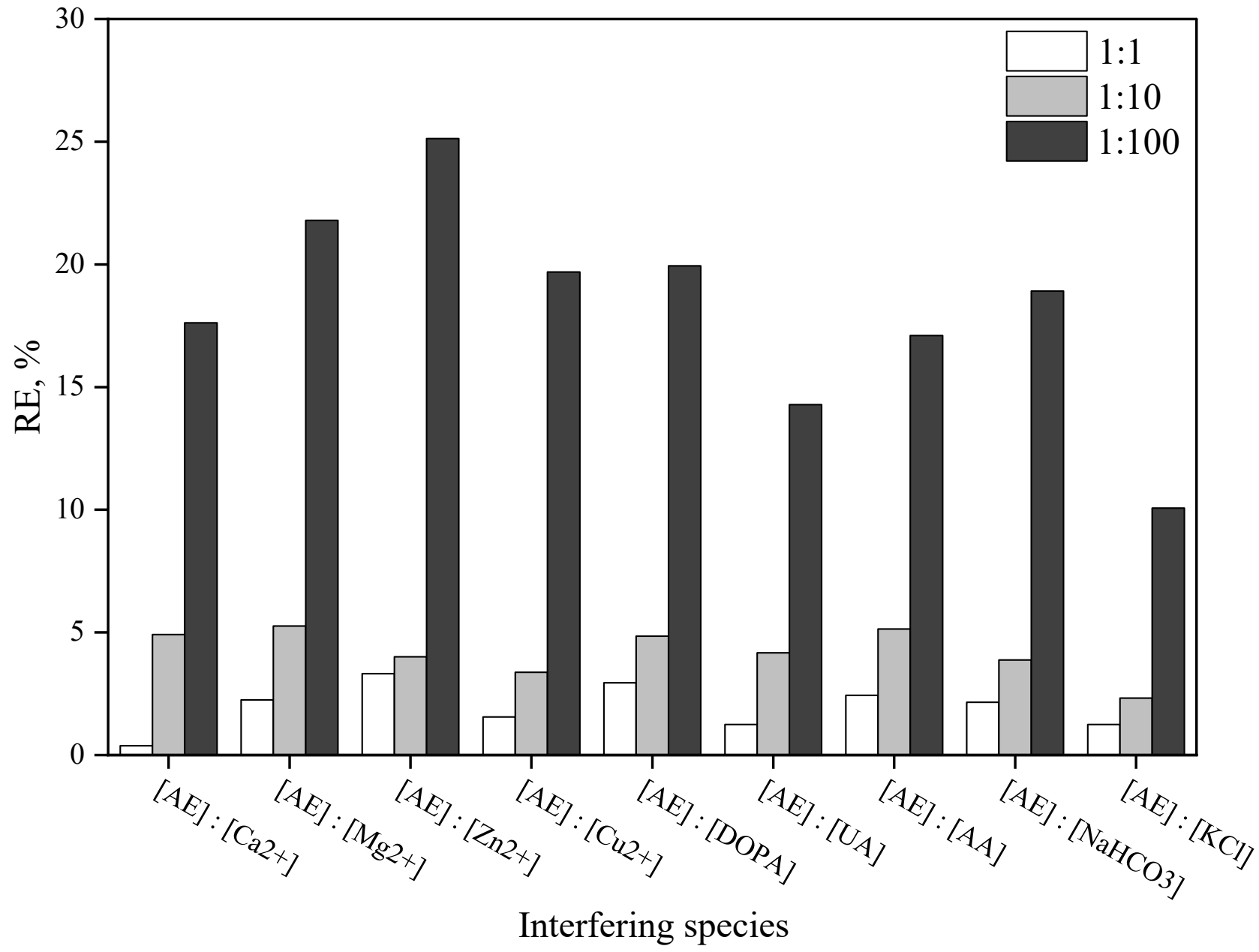

Fig. S4 Effect of interfering species (Ca^{2+}, Mg^{2+}, Zn^{2+}, Cu^{2+}, DOPA, UA, AA, $NaHCO_3$, and KCl) on DA/PdNPs/CPE in pH 7.0 PBS at 0.1 µM AE. (Molar ratios: 1:1, 1:10, and 1:100 [AE] : [interfering species]).

Table S3 Quantitative selectivity assessment of the DA/PdNPs/CPE sensor for AE.

Interfering Species	RE (%) [a] [1:1]	Selectivity Assessment	RE (%) [a] [1:10]	Selectivity Assessment	RE (%) [a] [1:100]	Selectivity Assessment
[AE]: [Ca^{2+}]	0.38	Excellent **(RE < 5-10%)**	4.91	Excellent	17.62	Unsatisfactory **(RE > 10%)**
[AE]: [Mg^{2+}]	2.25	Excellent	5.26	Excellent	21.80	Unsatisfactory
[AE]: [Zn^{2+}]	3.32	Excellent	4.00	Excellent	25.12	Unsatisfactory
[AE]: [Cu^{2+}]	1.55	Excellent	3.38	Excellent	19.69	Unsatisfactory
[AE]: [DOPA]	2.94	Excellent	4.85	Excellent	19.94	Unsatisfactory
[AE]: [UA]	1.25	Excellent	4.17	Excellent	14.28	Unsatisfactory
[AE]: [AA]	2.44	Excellent	5.14	Excellent	17.10	Unsatisfactory
[AE]: [$NaHCO_3$]	2.15	Excellent	3.88	Excellent	18.91	Unsatisfactory
[AE]: [KCl]	1.25	Excellent	2.32	Excellent	10.07	Unsatisfactory

[a] RE (%) = $[(\Delta i_p(\text{AE+Int}) - \Delta i_p(\text{AE})) / \Delta i_p(\text{AE})] \times 100$

Measured in 0.05 M PBS (pH 7.0) by SWV; all species at 0.1 µM AE (1:1, 1:10, and 1:100 ratio with AE)

Values represent the mean of triplicate measurements (n = 3)
